# The role of key gut microbial metabolites in the development and treatment of cancer

**DOI:** 10.1080/19490976.2022.2038865

**Published:** 2022-02-27

**Authors:** Kayla Jaye, Chun Guang Li, Dennis Chang, Deep Jyoti Bhuyan

**Affiliations:** NICM Health Research Institute, Western Sydney University, Penrith, NSW, Australia

**Keywords:** Gut microbiota, gut microbiome, gut metabolites, anticancer, bacteriocin, short-chain fatty acids (SCFA), phenylpropanoid, prenylflavonoids, ellagitannins, natural purine nucleoside, secondary bile acid, carcinogenesis

## Abstract

In recent years, the role of gut microbial metabolites on the inhibition and progression of cancer has gained significant interest in anticancer research. It has been established that the gut microbiome plays a pivotal role in the development, treatment and prognosis of different cancer types which is often mediated through the gut microbial metabolites. For instance, gut microbial metabolites including bacteriocins, short-chain fatty acids and phenylpropanoid-derived metabolites have displayed direct and indirect anticancer activities through different molecular mechanisms. Despite the reported anticancer activity, some gut microbial metabolites including secondary bile acids have exhibited pro-carcinogenic properties. This review draws a critical summary and assessment of the current studies demonstrating the carcinogenic and anticancer activity of gut microbial metabolites and emphasises the need to further investigate the interactions of these metabolites with the immune system as well as the tumour microenvironment in molecular mechanistic and clinical studies.

## Graphical abstract

**Figure:**
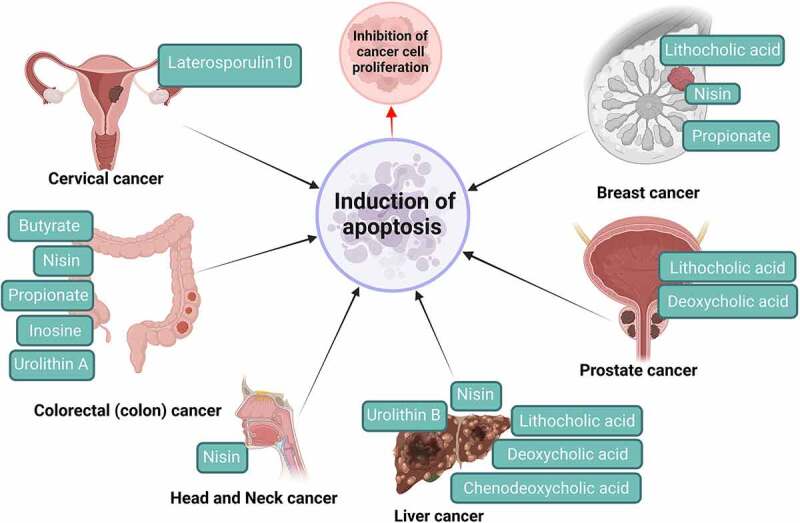

## Introduction

Over the past decade, the gut microbiome has been extensively investigated in the context of the maintenance of human health. Gut microbiota has been observed to maintain a mutually beneficial relationship with the host through modulation of gut homeostasis and the preservation of the epithelial barrier which are crucial for gut immunity ^1^. These microorganisms are important in the normal physiological function and structure of the host innate immune system, which has a number of implications on gut health.^2^ Emerging evidence has indicated that in addition to gut microbiota and their structural components, the myriad of metabolites produced by gut microbial communities also influence the host physiology and health by acting as signalling molecules and substrates for metabolic reactions.^3^ Our recent review has underlined the pivotal role of gut microbiota in the prevention, therapy and clinical outcome of the five most prevalent cancers while emphasising the direct and indirect impacts of gut microbial metabolites on tumours.^4^ Several studies in the literature have also demonstrated the potential effect of gut microbial metabolites in different diseases including cancer. This review provides a critical summary and assessment of the current studies performed to understand the role of the key gut microbial metabolites in cancer development, prevention, and treatment. This review also proposes several potential future directions in this emerging area of cancer research.

**Figure 1.**
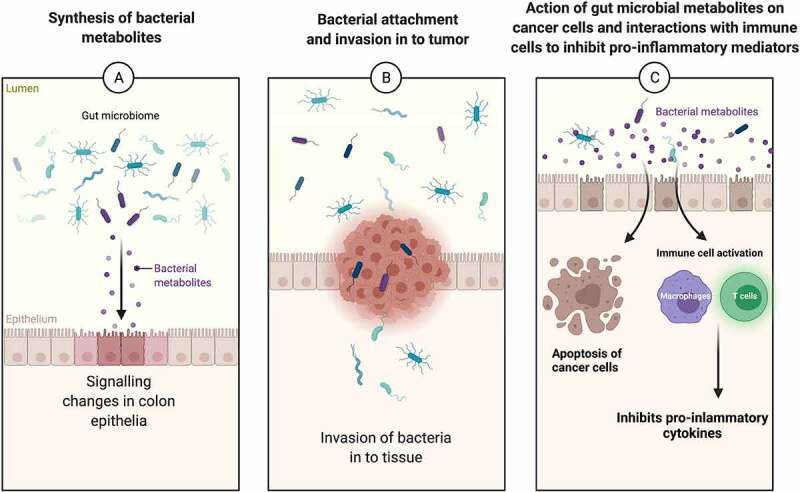
A schematic representation of (a) the signalling effect of gut microbial metabolites on the colon epithelium, (b) initiating an invasion of the bacterial species into the colorectal cancer tissue.^6^ In addition, (c) gut microbial metabolites induce apoptosis in cancer cells and activate immune cells to inhibit pro-inflammatory cytokines.^5^

Gut metabolites have been shown to exhibit a variety of pro- and anti-carcinogenic effects on cancer. Recent reviews demonstrated the dual role of gut metabolites in promoting as well as preventing colorectal cancer (CRC)^5,6^ (Figure 1). It has also been suggested that the cumulative effects of the microbial metabolites should be considered to predict and prevent the progression of CRC.^5^ Furthermore, it has been postulated that the increase in CRC risk is due to an imbalance between health-promoting metabolites such as butyrate and potentially carcinogenic metabolites including secondary bile acids (BAs).^7^ In addition to the pro-carcinogenic activity of secondary BAs, microbial metabolism may also highlight the role of dietary fat (which increases the synthesis of BAs in the liver) on colon cancer progression as observed in rural African and African American populations.^7^ The influence of diet on colonic health was further observed in a study that assessed the impact of a high-protein and low-carbohydrate diet on the metabolic profile in the colon.^8^ The author reported that the high-protein and reduced-carbohydrate diet had a detrimental effect on colon health by causing an observable decrease in faecal anticancer metabolites and an increase in concentrations of carcinogenic metabolites, constituting an increased risk of CRC in individuals who adhere to this diet long-term.^8^ Further evidence supporting this trend has led to the understanding that protein fermentation in the distal colon has detrimental effects on host health by producing toxic ammonia, amines, phenols and sulfides, in comparison to carbohydrate fermentation which generates health benefitting short-chain fatty acids (SCFAs).^9^ The review by Windey et al. in 2012 ^9^ also indicated that a diet rich in meat increases fermentation of proteins in addition to increased intake of fat, heme and heterocyclic amines, which may collectively contribute to the higher prevalence of CRC in Western society. However, more in-depth mechanistic studies are necessary to understand the relationship between protein fermentation and gut health in relation to CRC.

**Figure 2.**
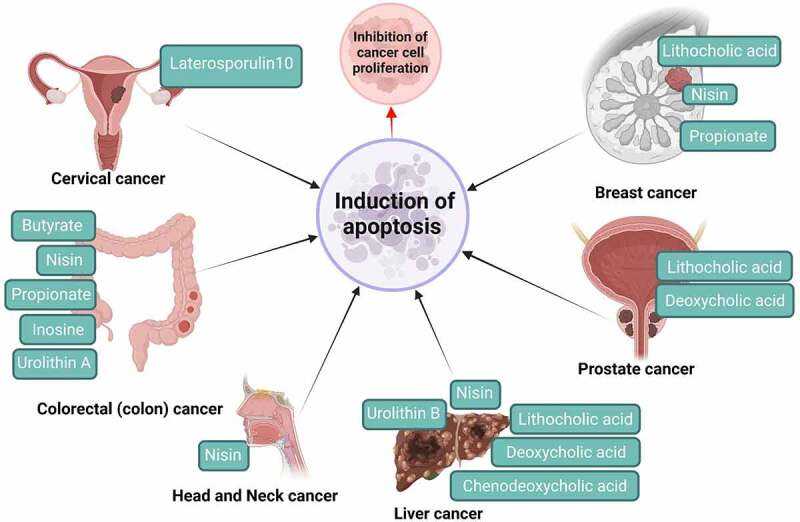
An overview of gut microbial metabolites and their action on cervical, breast, colon, prostate, head and neck, and liver cancers. These metabolites inhibit the proliferation and survival of cancer cells or tumours *via* induction of apoptosis.

Prebiotic and probiotic-based strategies targeted at improving systemic health has led to an increased interest in the role of SCFAs including butyrate, acetate, and propionate which are the by-products (secondary metabolites) of carbohydrate fermentation by the gut microbial communities.^10^ Whilst SCFAs exhibit cancer-protective properties during dietary fibre fermentation, secondary BAs, at high physiological levels in the colon, elicit an opposite effect through induction of colonic inflammation.^10,11^ A high abundance of secondary BAs correlates with a high-fat diet, in which exposure to BAs can generate reactive oxygen species and disrupt the cell membrane and mitochondria.^12^ Other bacterial metabolites such as bacteriocins have been used safely in the food industry as a food preservative and are emerging as potential therapeutic agents against colon, head and neck, breast, brain, skin, and liver cancers.^13–20^ Nisin is a well-researched bacteriocin that has demonstrated cytotoxic effects on CRC and head and neck squamous cell carcinoma both *in vitro* and *in vivo* mediated *via* induction of apoptosis.^13,16^ Despite the reported potential pro- and anticancer activities, further animal and clinical studies are required to develop a better understanding of the role of gut metabolites in the progression as well as prevention of cancer and to develop precision anticancer therapies.

Phenylpropanoids are a diverse family of plant secondary metabolites synthesized from the amino acids- phenylalanine and tyrosine. Plant-based diets consisting of phenylpropanoids have been suggested to improve human health. However, specific microbial species have the capacity to ferment the three aromatic amino acids (AAAs)- phenylalanine, tyrosine and tryptophan to phenylacetic acid (PAA) and 4‐hydroxylphenylacetic acid (4‐hydroxyPAA) indicating that protein fermentation is the probable source of phenylpropanoid-derived by-products within the colon.^21^ Although the study by Russell et al. in 2013^21^ indicated that gut microbiota can ferment proteins to produce major phenylpropanoid‐derived metabolites, the same research group earlier in 2011 suggested that a high-protein and low total carbohydrates and fibre diet can significantly decrease faecal cancer-protective metabolites and increase the concentrations of hazardous metabolites.^8^

## Bacteriocins

**Figure 3.**
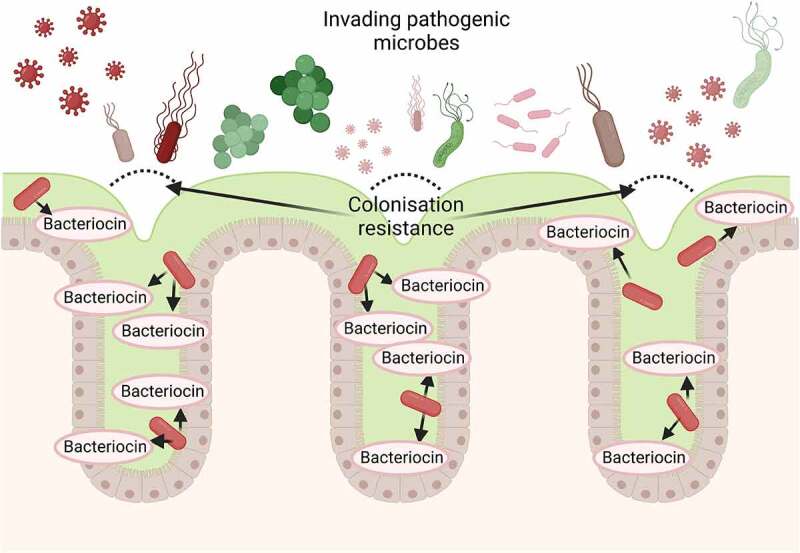
A simplified diagrammatic representation of the ‘colonisation resistance’ process initiated by lactic acid bacteria through the production of bacteriocins on the colonic epithelial surface to prevent the colonisation of pathogenic microbes.

Bacteriocins are cationic peptides produced by certain probiotic bacteria in the gut through ribosomal activity and structurally classified as bacterial antimicrobial peptides.^22–24^ Bacteriocins have demonstrated significant inhibition of other bacteria such as antibiotic-resistant strains with narrow to broad-spectrum activity.^22^ These antimicrobial peptides can also inhibit pathogenic bacteria in the gut^25^ and therefore, are important in maintaining gut homeostasis. Lactic acid bacteria is one of the most significant sources of bacteriocins, especially the genus *Enterococcus* belonging to the phylum Firmicutes.^26,27^ A clinical study profiled the prevalence of bacteriocin production by the pathogenic *Escherichia coli* strains in CRC patients.^28^ The clinical trial included 30 patients with colorectal cancer, 30 patients with colorectal adenoma, and 20 healthy controls, and evaluated the bacteriocins- colicin Ia, colicin M, microcin mH47, microcin mV, and microcin mM.^28^ This study observed that advanced stage CRC patients presented with more virulent strains of *E. coli*, and this correlated with increased production of bacteriocins in comparison to less advanced stage diagnoses.^28^ Bacteriocins have exhibited significant cytotoxicity against cancer cells *in vitro* (Table 1) and low cytotoxicity towards normal intestinal epithelial cells.^18^ In addition, the anticancer activity of bacteriocins is also attributed to their capacity to inhibit the colonisation of competing pathogenic bacterial strains in a phenomenon known as ‘colonisation resistance’ (Figure 3), as well as the immunomodulation of the gut microbial composition.^66^ The cellular membrane is the primary target of bacteriocins in eukaryotic cells, in which bacteriocins increase the expression of negatively charged cell-surface molecules on cancer cells and encourage cytotoxicity.^67^ The proposed mechanisms of action of this activity are the induction of apoptotic cell death and the depolarization of the cell membrane leading to changes in cell membrane permeability, indicating a non-receptor-modulated process.^67^ In addition to selective cytotoxicity against cancer cells, the non-immunogenic and biodegradable nature of bacteriocins make them a promising candidate for novel anticancer therapy.^67^ Therefore, the possibilities to modulate the production of bacteriocins by the probiotic bacteria in the gut as well as their bioengineering have also been proposed for their clinical applications.^22^

**Table 1.** Key gut microbial metabolites and their action against cancer *in vitro* and *in vivo.*

Metabolite	Type of study	Type of cancer	Type of cell line/ animal	Details of the clinical study	Effect	Type of assay	Molecular mechanisms of action	References
**Bacteriocins**								
Nisin	*In vitro*	Colon cancer	SW480 epithelial-like colon cancer cells	N/A	Cytotoxic activity (IC_50_ = 600 µM/mL).	MTT, PCR, and Western blot.	Increased apoptotic index in cells *via* the intrinsic apoptotic pathway.	^13^
	*In vitro*	Colon cancer	SW480 colorectal cancer cells and NIH 3T3 mouse embryo fibroblast cells.	N/A	Inhibited cell proliferation of the SW480 cells (IC_50_ = 250 µM/mL).	MTT and PCR.	Induced apoptotic pathway *via* an increased expression of caspase 3 and 9, and an increased ratio of Bax/Bcl2.	^29^
	*In vitro*	Gastrointestinal cancer, liver cancer, and blood cancer	AGS and KYSE-30 gastrointestinal cancer cells, HepG2 hepatic cancer cells, and K562 blood cancer cells.	N/A	Exhibited cytotoxicity and inhibited cell growth of AGS (IC_50_ = 61 ± 3 µM/mL), KYSE-30 (IC_50_ = 130 ± 5 µM/mL), HepG2 (IC_50_ = 95 ± 3 µM/mL), and K562 (IC_50_ = 146 ± 5 µM/mL) cells.	MTT, Neutral Red, Ethidium Bromide /Acridine Orange staining and fluorescein isothiocyanate imaging.	Induced the apoptotic pathway, which was also supported by observed morphological changes in cancer cells.	^30^
	*In vitro*	Head and neck cancer	UM-SCC-17B, UM-SCC-14A and OSCC-3 human squamous carcinoma, HSC-3 human tongue squamous carcinoma cells.	N/A	Nisin ZP (95% purity) exhibited strong anticancer effects.	CyQUANT NF cell proliferation assay, Orasphere assay, flow cytometry, ethidium bromide and acridine orange staining, *in vitro* sprout assays and Western blot analyses.	Induced apoptosis in cancer cells *via* calpain-dependent pathway (with caspase 3, 8 and PARP cleavage) but not in human oral keratinocytes. Reduced vascular sprout formation and inhibited cell proliferation.	^16^
	*In vivo*	Head and neck cancer	Oral cancer floor-of-mouth mouse model xenografted with the UM-SCC-17B cells.	N/A	Nisin ZP (95% purity): a dose of 800 mg/kg body weight/day was administered. Inhibited tumorigenesis.	Measurement of tumour volume and immunohistochemical analyses.	Nisin ZP reduced tumorigenesis *in vivo* and long-term treatment with nisin ZP extended survival of the mice with normal organ histology and without inflammation, fibrosis and necrosis.	^16^
	*In vitro*	Breast cancer	MCF7 human breast carcinoma cells	N/A	Strong cytotoxicity with an IC_50_ value of 5 μM/mL.	MTT and cell morphology analysis using a microscope.	Induced apoptosis, cell cycle arrest and calcium influx.	^14^
	*In vitro*	Brain cancer	SW1088 human astrocytoma cells	N/A	Inhibited cell proliferation with IC50 value of 50 μg/mL, 75 μg/mL, and 50 μg/mL at 24, 48, and 72 hours, respectively.	MTT and annexin V-FITC /propidium iodide staining.	Induced apoptotic processes and cell death, down-regulated cell viability dose-dependently.	^20^
	*In vitro*	Skin cancer	A375 melanoma cells	N/A	Cytotoxicity with an IC_50_ value of 180 μM/mL.	MTT, LDH and flow cytometry.	Cytotoxic against cancer cells, and low cytotoxicity against non-malignant cells. Activated apoptotic pathway, causing disruption to the cell membrane.	^17^
	*In vitro*	Breast and liver cancer	MCF7 human breast adenocarcinoma and HepG2 liver carcinoma cells.	N/A	Cytotoxicity against the MCF-7 (IC_50_ = 105.46 μM/mL) and the HepG2 cells (IC_50_ = 112.25 μM/mL).	MTT and cell morphology analysis using light microscopy.	Strong haemolytic activity against eukaryotic cells and increased the permeability of the phospholipid bilayer.	^19^
	*In vitro*	Colon cancer	HT29 and Caco-2 colorectal adenocarcinoma cells.	N/A	Cytotoxicity against the HT29 (IC_50_ = 89.9 μM/mL) and Caco-2 (IC_50_ = 115 μM/mL) cells.	MTT, neutral red dye uptake assay, Haemolysis assays and trans-epithelial electrical resistance assay.	Demonstrated significant haemolysis and lower selective toxicity and cytotoxicity within gastrointestinal cells.	^18^
	*In vitro*	Head and neck cancer	UM-SCC-17B squamous carcinoma cells	N/A	Significant cancer cell growth inhibition observed.	Fluo-4 based calcium concentration assay, ELISA apoptotic assay, cell cycle analysis by flow cytometry, gene array analyses and Western blot	Decreased tumorigenesis through induction of apoptosis *via* induction of CHAC1 expression. Nisin reduced HNSCC cell proliferation by arresting cells in the G_2_ phase.	^15^
	*In vivo*	Head and neck cancer	Oral cancer floor-of-mouth mouse model: Athymic nude mice; NCr-nu/nu strain xenografted with HNSCC cells.	N/A	Reduced HNSCC tumorigenesis and significant reduction of tumour volume.	*In vivo* toxicity assay, measurement of tumour volume.	Reduction of tumour volume was mediated *via* induction of CHAC1 expression (suppression significantly increased tumour volume).	^15^
Laterosporulin10 (LS10)	*In vitro*	Cervical cancer, breast cancer, bone cancer, and lung cancer	HeLa cervical adenocarcinoma, MCF7 breast adenocarcinoma, HT1080 fibrosarcoma, H1299 lung cancer, and HEK293T human embryonic kidney cells.	N/A	Exhibited cytotoxicity against the cancer cells (HeLa = 80%, MCF7 = 40% and HT1080, HEK293T and H1299 = 20% cell growth inhibition).	MTT, LDH release assay, electron microscopy and flow cytometry.	Caused apoptotic and necrotic cancer cell death at various concentrations, with dose-dependent cytotoxicity, and release of lactate dehydrogenase from the cancer cells.	^31^
**Short-chain fatty acids**								
Butyrate	*In vitro*	Colon cancer	HCT8 human colonic adenocarcinoma cells	N/A	Inhibited cell proliferation (IC_50_ = 2 mM/mL before G protein-coupled receptor 43 (GPR43) expression and 0.8 mM/mL after GPR43 expression).	MTS, immunohistochemistry, Promega Dual-Luciferase (R) Reporter (DLR[TM]) Assay System, RT-PCR, Western blot, cAMP-Glo assay, flow cytometry, colony formation assay and immunoblot.	Intracellular actions involving the inhibition of histone deacetylase. Post-GPR43 expression, exhibited increased apoptotic cell death and inhibited cell proliferation through cell cycle arrest at the G_0_/G_1_ phase.	^32^
	*In vitro*	Colorectal cancer	HCT116 human colorectal cancer cells	N/A	Inhibited cell growth dose-dependently, exhibiting 100% growth inhibition at 5 mM after 24, 48, and 72 h.	MTT, Western blot, ELISA, and PCR.	Treatment inhibited cell proliferation of HCT116 cells both dose- and time-dependently, and induced apoptosis time-dependently. Induction of apoptosis through activation of caspase-3 in a time-dependent manner with 1 mM butyrate. Apoptosis was mediated *via* modulation of Bax and Bcl-2 expression (increased Bax/Bcl-2 ratio). Deactivated mTOR/S6K1 (mammalian target of rapamycin/ribosomal protein S6 kinase β‑1) signalling potentially *via* inhibition of SIRT1 (silent mating type information regulation 2 homolog).	^33^
	*In vitro*	Colorectal cancer	WiDr and C2BBe1 human colorectal adenocarcinoma cells, LS1034 chemoresistance human colorectal carcinoma cells, and HFF1 fibroblast cells.	N/A	At 48 h of exposure, metabolic activity and proliferation were impacted of the C2BBe1 (IC_50_ = 14.4 mM), WiDr (IC_50_ = 2.8 mM), and LS1034 (IC_50_ = 6.8 mM) cells.	MTT, annexin-V/propidium iodide (AV/PI) incorporation cell viability assay, flow cytometry, immunofluorescence, and Western blot.	Reduced metabolic activity and inhibited cell proliferation in cancer cells dose-dependently. Induced cell death *via* apoptotic and necrotic processes. Cell cycle arrest occurred at G_0_/G_1_ stage for LS1034 and WiDr, and G_2_/M for C2BBe1 and WiDr. Treatment also increased the Bax/Bcl2 ratio and p21 expression in all cell lines, inhibited the β-catenin expression and decreased p53 expression (in the LS1034 and WiDr cells) and P-glycoprotein activity (in the chemoresistant LS1034 cells). Butyrate improved the efficacy of standard chemotherapeutic drug Irinotecan through reduction of the IC_50_ values against all tested cancer cell lines. Butyrate alone and in combination with Irinotecan was demonstrated to confer anticancer effect independently of P53 status of the cancer cells.	^34^
	*In vivo*	Colorectal cancer	Balb/c nu/nu mice xenografted with WiDr cells.	N/A	Butyrate in combination with Irinotecan inhibited tumour growth.	-	-	^34^
	*In vitro*	Colorectal cancer	HCT116 and LoVo human colorectal cancer cells.	N/A	The IC_50_ values were not reported.	CCK-8, ELISA, Western blot, PCR, and flow cytometry.	Butyrate (2 mM) inhibited glucose transport and glycolysis of colorectal cancer cells glucose uptake *via* reduction of membrane GLUT1 content and cytoplasmic G6PD which was regulated by the GPR109a-AKT signalling pathway. Enhanced the apoptosis efficacy of 5-FU against the cancer cells through impaired DNA synthesis efficiency (*via* modulation of the AKT signalling pathway).	^35^
	*In vitro*	Breast cancer and leukaemia	MCF7 breast adenocarcinoma and HL-60 promyelocytic leukaemia cells.	N/A	Both cell lines internalised the DiO-tagged cholesteryl butyrate solid lipid nanoparticles 0.50 mM/mL in more than 80% of the whole cell population.	WST-1, PCR and flow cytometry.	In the HL-60 cell line, delivery of butyrate by solid lipid nanoparticles increased anticancer activity and potential. In the MCF-7 cell line, it inhibited cell proliferation *via* the p53 pathway.	^36^
Sodium butyrate	*In vitro*	Breast cancer	MCF7 and MDA-MB-468 breast adenocarcinoma cells.	N/A	Exhibited cytotoxicity time- and dose-dependently. Reduced cell viability of MCF7 cells by 40% at 10 mM and 27% at 5 mM. Similarly, reduced cell viability of the MDA-MB-468 cells by 43% at 10 mM and 30% at 5 mM.	MTT and flow cytometry.	Induced cell cycle arrest and apoptotic cell death. This correlated with an increase in reactive oxygen species (ROS) and mitochondrial membrane potential modulatory action.	^37^
	*In vitro*	Breast cancer	MCF7 human breast adenocarcinoma cells	N/A	Inhibited cell proliferation dose- and time-dependently. Reduction of cell viability 48 h following treatment.	CCK-8 cell viability assay and Western blot.	Induced apoptotic cell death and led to significant changes in cell morphology after treatment.	^38^
	*In vitro*	Breast cancer	MCF7, T47-D, BT-20, and MDA-MB-231 breast cancer cells.	N/A	Reduction in cell number and growth in all cell lines tested ranging from 20–75%.	Western blot and PCR.	Induced cell cycle arrest in the G_1_ phase and apoptosis in the MCF&, T47-D and BT-20 cell lines, and arrested the MDA-MB-231 cells in the G_2_/M phase.	^39^
	*In vitro*	Breast cancer	MCF7 human breast adenocarcinoma cells	N/A	Inhibited cell proliferation dose-dependently (IC_50_ = 1.26 mM/mL).	MTT and flow cytometry.	Higher concentrations increased the level of apoptosis. Cell cycle arrest in the G_1_ growth phase was also observed.	^40^
	*In vitro*	Colorectal cancer	HCT116 and HT-29 human colorectal cells, and CCD841CoN human normal colon cells.	N/A	Inhibited cell viability in the HCT116 cancer cells (IC_50_ = 3.189 mM) and HT-29 cancer cells (IC_50_ = 3.338 mM).	MTT, ELISA, and Western blot.	Exhibited synergistic activity with alkylresorcinol C21 against the colon cancer cells (C21 at 40 μM and sodium butyrate at 1–4 mM). The combined treatment (C21 and sodium butyrate) upregulated cleaved Poly(ADP-ribose) polymerase (PARP), cleaved caspase 3, p53 upregulated modulator of apoptosis (PUMA), cytochrome C, lipid-conjugated membrane-bound form of microtubule-associated protein 1A/1B-light chain 3 (LC3-II), and C/EBP homologous protein (CHOP) expressions indicating the induction of apoptosis, autophagy, and ER stress pathways in the cancer cells. The combination showed less toxicity against the CCD841CoN human normal colon cells.	^41^
	*In vitro*	Colorectal cancer	Caco-2 and HT-29 human colorectal cancer cells.	N/A	Significant modulation of cell viability was detected, however, no IC_50_ was reported.	MTT, Alkaline phosphatase, Immunoblotting, Acetyl-coenzyme A and α-ketoglutarate, Immunofluorescence and immunoprecipitation assays.	Sodium butyrate (4 mM) suppressed cell proliferation, increased cell differentiation, and induced apoptotic cell death. Increased protein contents and activities of isocitrate dehydrogenase 1 and pyruvate dehydrogenase in colorectal cancer cells. Upregulated αacetyl-CoA and α-ketoglutarate and enhanced histone acetylation and DNA demethylation in the promoter of DNA mismatch repair gene.	^42^
Propionate	*In vitro*	Colon cancer	HCT8 human colonic adenocarcinoma cells	N/A	Inhibited cell proliferation (IC_50_ = 5 mM/mL (before GPR43 expression and 2 mM/mL after GPR43 expression).	MTS, immunohistochemistry, Promega Dual-Luciferase (R) Reporter (DLR[TM]) Assay System, RT-PCR, Western blot, cAMP-Glo assay, flow cytometry, colony formation assay and immunoblot.	Intracellular actions involving the inhibition of histone deacetylase. Post-GPR43 expression, exhibited increased apoptotic cell death and inhibited cell proliferation through cell cycle arrest at the G_0_/G_1_ phase.	^32^
Sodium propionate	*In vitro*	Breast cancer	MCF7 human breast adenocarcinoma cells	N/A	Inhibited cell proliferation dose-dependently (IC_50_ = 4.5 mM/mL).	MTT and flow cytometry.	Inhibited cell growth and cell proliferation in a dose-dependent manner and caused a blockage in stage G_1_ of the cell cycle. Induced apoptotic cell death dose-dependently.	^40^
	*In vitro*	Lung cancer	H1299 and H1703 non-small cell lung carcinoma cells.		Inhibited cell proliferation, however, no IC_50_ values were reported.	Western blot and PCR.	At 10 mM, sodium propionate suppressed cell growth and proliferation. Induced cell cycle arrest in the G_2_/M phase leading to apoptotic cell death. Upregulated the expressions of p21 and survivin leading to suppression of cell proliferation.	^43^
**Phenylpropanoid-derived metabolites**								
9,9^′^,-*O*-feruloyl-(−)-secoisolaricinresinol	*In vitro*	Breast cancer	MCF-7 human breastcarcinoma cells	N/A	Strong cytotoxic activity with an EC_50_ value of 3.9 μg/mL.	Sulforhodamine B (SRB)assay	No reported	^44^
Verbascoside	*In vitro* and *In vivo*	Oral cancer	HN4 and HN6 human oral squamous cell carcinoma cells. BALB/c nude female mice xenograft oral squamous cell carcinoma model (HN4 and HN6 oral squamous tumour).	N/A	Decreased cell proliferation. Cell viability = 75%. Strongly inhibited growth and lung metastasis of implanted tumour cells.	MTT, acridine orange/ethidium bromide, flow cytometry, TUNEL assay, wound-healing assay, RT-PCR, and Western blot. Blood analysis and histological examination, and H&E staining.	*In vitro* Induced apoptotic cell death and inhibited cell migration of the HN4 and HN6 cells. *In vivo* Demonstrated biocompatibility without adverse effects, with increased apoptotic cell death and decrease in cancer cell survival. Decreased nuclear factor (NF)-κB activation leading to suppression of mRNA and protein expression of matrix metalloproteinase-9 thereby inhibiting tumour cell metastasis. Downregulated anti-apoptotic Bcl-2/Bcl-XL expression and upregulated apoptotic Bax expression.	^45^
	*In vitro, in vivo* and human tissues	Colon cancer	HCT-116, HT-29, LoVo and SW620 human colorectal cancer cells. BALB/c nude male mice xenograft of the HCT-116 cells.	N/A	Significant inhibition of cell proliferation in a dose- and time-dependent manner in all studied cell lines- HCT-116 (IC_50_ = 63.51 μM/L), LoVo (IC_50_ = 43.96 μM/L), HT-29 (IC_50_ = 66.68 μM/L) and SW620 (IC_50_ = 29.05 μM/L). Inhibited cell proliferation and decreased tumour volume by 63.75% in high-dose (100 μM) and by 48.41% in low-dose (25 μM)	CCK-8, flow cytometry,Western blot, and the measurement of tumour size.	*In vitro* The flow cytometry method observed early and late-stage apoptosis, as well as inhibition of cell proliferation. HIPK2 regulated the phosphorylation of p53, as well as the concentration of Bcl-2 and Bax in these cancer cells. *In vivo* Enhanced the expression of pro-apoptotic HIPK2, p53, and Bax proteins in tumours, but decreased expression of anti-apoptotic protein Bcl-2, in a dose-dependent manner. Human tissue In human colorectal cancer tissues, the expression of HIPK2 was significantly lower compared to normal tissues. The expression of HIPK2 in human colorectal cancer significantly correlated with the degree of differentiation.	^46^
	*In vitro*	Gastric cancer	MKN45 gastric adenocarcinoma cells	N/A	Exhibited cytotoxicity (IC_50_ = 17.8 ± 7.2 μg/mL).	Trypan blue assay, PCR and flow cytometry.	Induced cell cycle arrest at the sub-G_0_/G_1_ and G_2_/M phases. Additionally, mediated cell differentiation and apoptotic processes, which may be a result of inhibiting telomerase activity in cancer cells.	^47^
**Prenylflavonoids**								
8-prenylnaringenin	*In vitro*	Colorectal cancer	Caco-2 human colorectal adenocarcinoma cells, HT115 human colorectal carcinoma cells, and MRC-5 human fetal lung fibroblast cells.	N/A	A significant decrease in the number of viable cells was observed at 40 μM (*p* < 0.01) with a reduction of 25% in cell viability of the Caco-2 cells after 24 h pre-incubation.	MTT, DNA content, Comet, Matrigel and flow cytometry.	Decreased DNA damage in the Caco-2 cells induced by exogenous H_2_O_2_ at concentrations up to 40 μM. Significantly increased the sub-G_1_ and G_1_ phases, marginally enhanced the S-phase component with no impact on the G_2_/M phase at all concentrations (12.5 μM, 25 μM, and 50 μM) in the Caco-2 cells. Led to significant reductions of the HT115 cell invasiveness at 5, 10, and 20 μM with up to 46% decrease.	^48^
	*In vitro* and *in silico*	Melanoma	SK-MEL-28 and BLM metastatic melanoma cancer cells.	N/A	Inhibited cell viability and growth at 8-PN concentrations of between 50–100 µmol/L.	HDAC inhibitor screening assay, HDAC inhibition profiling assay, cell proliferation assay, real-time cell monitoring assay, Western blot, flow cytometry, and human proteome profiler apoptosis antibody array analyses.	*In silico*, fit into the binding pocket of HDAC enzymes- 2, 4, 7 and 8 (binding to the zinc ion of their catalytic centre). *In vitro*, Inhibited cell proliferation and viability dose-dependently, induced hyperacetylation of histone complex H3, and apoptosis. This activity occurred *via* down-regulation of mTOR-specific pS6 protein *via* the pERK/pP90 pathway.	^49^
	*In vitro*	Colon cancer	HCT-116 colorectal cancer cells	N/A	Demonstrated strong inhibitory activity against the HCT-116 cells with an IC_50_ value of 23.83 ± 2.9 μg/mL after 48 h.	MTT, acridine orange/propidium iodide staining, and caspase luminescence-based assays.	Inhibited cell proliferation and induced intrinsic and extrinsic pathway-mediated apoptotic cell death. Cell cycle arrest was induced at the G_0_/G_1_ phase.	^50^
	*In vitro*	Breast cancer	MCF7 and MDA-MB-231 human breast cancer cells.	N/A	Inhibited cell viability of MCF7 cells at a concentration of 10 uM of 8-PN.	Western blot, Hoechst 33,258 staining, and flow cytometry	Inhibited cell proliferation and induced apoptotic cell death. Inhibited the growth of estrogen-responsive cells *via* interference with the estrogen receptor-associated PI3K molecular pathway. 8-PN also modulated levels of cyclin D1 expression.	^51^
Dihydroxanthohumol	*In vitro*	Colorectal and liver cancers	HCT116 and HT29 human colon cells, and HepG2 and Huh7 hepatocellular carcinoma cells.	N/A	Inhibited cell proliferation in HCT116 cells (IC_50_ = 28.7 uM), HT29 cells (IC_50_ = 31.4 uM), HepG2 cells (IC_50_ = 21.7 uM), and Huh7 cells (IC_50_ = 32.5 uM).	SRB assay and flow cytometry.	Inhibited cell proliferation across all cell lines. Significantly induced apoptosis dose-dependently *via* caspase activation.	^52^
**Ellagitannins**								
Urolithin A	*In vitro*	Colorectal cancer	Caco-2, HT-29, and HCT-116 human colon cancer cells, and CCD18-Co non-tumorigenic colon cells.	N/A	Reduced colony formation capacity, however, no IC_50_ values were reported. The highest tested concentration of urolithin A was 10 μM.	MTT, clonogenic, flow cytometry, Senescence-associated β-galactosidase, Western blot and LC-MS assays.	Dose-dependent anti-clonogenic effect through the increase of the senescence-associated β–galactosidase activity. Senescence of the HCT-116 cells (p53-wild type) with elevated p53 and p21^Cip1/Waf1^ expression. Reduced the colony formation capacity in the HCT-116 cells. Induced cell cycle arrest at the G_0_/G_1_ and G_2_/M phases in the HCT-116 and Caco-2 cells, respectively.	^53^
	*In vitro*	Colorectal cancer	Caco-2, HT-29, and SW480 human colon cancer cells.	N/A	Exhibited cytotoxicity but the IC_50_ values were not reported. Two concentrations (100 and 50 μM) and two time points (24 and 48 h) were tested.	MTT, Trypan blue, flow cytometry and HPLC-MS assays.	Inhibited cell proliferation in a concentration- and time-dependent manner. Induced cell cycle arrest at the G_2_/M and S phases in both cell lines.	^54^
	*In vitro*	Colorectal cancer	Caco-2, HT-29, and SW480 human colon cancer cells.	N/A	Modulated cell viability at 72 h in Caco-2 cells (IC_50_ = 32.50 uM), HT-29 cells (49.92 uM), and SW480 cells (35.92 uM).	MTT, flow cytometry, annexin V/PI, and Western blot.	Urolithin A (0.8–400 μM) alone inhibited cell proliferation in a time-dependent manner (48 and 72 h). Induced cell cycle arrest at the G_2_/M phase in the Caco-2 and SW480 cells at 20 μM *via* the upregulation of cyclin A and B1. Induced apoptotic cell death. Co-treatment with 5-FU and 5′DFUR decreased their respective IC_50_ values and arrested the cell cycle at the G_2_/M phase together with a slight enhancement of caspases 8 and 9 activations.	^55^
	*In vitro*	Colorectal cancer	HCT116 human colon carcinoma cells	N/A	Inhibited growth of HCT116 cells (IC_50_ = 19.6 uM at 72 h) and exhibited synergy with oxaliplatin.	Flow cytometry and Western blot.	Inhibited cell growth by >50%. Elicited p53-dependent and -independent signals that contribute to this inhibition. Induced cell cycle arrest in the G_2_/M phase (p53-independent). Reduced glycolytic potential (*via* the TP53-induced glycolytic regulatory phosphatase axis). Urolithin A interacted synergistically with oxaliplatin with combinatorial indices of <1 at all tested combinations.	^56^
Urolithin B	*In vitro*	Liver cancer	HepG2, Bel7402, Huh7 human hepatocellular cancer cells, and LO2 immortalised normal liver cells.	N/A	Inhibited cell proliferation in the HepG2 cells (IC_50_ = 15 uM) at 72 h.	Flow cytometry, CCK-8, colony formation, luciferase activity, PCR, immunoblotting and immunofluorescence assays.	Inhibited the growth of hepatocellular cancer cells *via* cell cycle arrest and apoptosis and demonstrated low toxicity towards normal liver cells. Induced cell cycle arrest at the G_0_/G_1_ phase in the HepG2 cells and at the S phase in the Bel7402 cells. Increased phosphorylated β-catenin expression and blocked its translocation from cytoplasm to the nucleus, therefore, inactivating Wnt/β‐catenin signalling.	^57^
	*In vivo*	Liver cancer	Nude mice xenografted with HepG2 cells.	N/A	Urolithin B (40 mg/kg) suppressed tumour growth.	Immunohistochemistry and the measurement of tumour size.	Reduced Ki-67, a classical marker of cell proliferation in the tumour.	^57^
	*In vitro*	Colorectal cancer	Caco-2, HT-29, and SW480 human colon cancer cells.	N/A	Exhibited cytotoxicity but the IC_50_ values were not reported. Two concentrations (100 and 50 μM) and two time points (24 and 48 h) were tested.	MTT, Trypan blue, flow cytometry and HPLC-MS assays.	Inhibited cell proliferation in a concentration- and time-dependent manner. Induced cell cycle arrest at the S phase (Caco-2 and HT-29 cells) with no effect on the cell cycle of SW480 cells.	^54^
**Natural purine nucleoside**								
Inosine	*In vivo*	Bladder cancer	Germ-free *Rag1*-deficient mice injected with the MB49 human bladder cancer cells.	N/A	No modulation to cell viability was detected, however, reduced tumour cell size.	Flow cytometry, tumour size and Q-PCR	Promoted immunotherapy response in mice. The proposed mechanism of action was enhancing the function of anti-CTLA-4 to increase infiltration of IFN-ɣ^+^CD4^+^ and IFN-ɣ^+^CD8^+^ T-cells into the tumour, as well as reducing overall tumour weight when administered with CpG (DNA oligonucleotides containing unmethylated deoxycytidylyl-deoxyguanosine dinucleotides) as a combination therapy.	^58^
	*In vivo*	Melanoma	Germ-free *Rag1*-deficient mice injected with the B16-F10 melanoma cancer cells.	N/A	No modulation to was cell viability detected, however, reduced tumour cell size.	Flow cytometry, tumour size and Q-PCR	Promoted immunotherapy response in mice. The proposed mechanism of action was perturbation to the ability of anti-CTLA-4 to modulate anticancer immune processes.	^58^
	*In vivo*	Intestinal cancer	Msh2LoxP/LoxP Villin-Cre mice were used to simulate intestinal carcinogenesis.	N/A	No modulation to cell viability was detected, however, reduced tumour cell size.	Flow cytometry, tumour size and Q-PCR	Promoted immunotherapy response in mice. The proposed mechanism of action was increasing the activation of a cDC-dependent T_H_1 cell circuit in the host, which enhanced the overall effect of the immune checkpoint blockade (ICB) therapy.	^58^
	*In vivo*	Colon cancer	Germ-free and specific-pathogen-free mice injected with the MC38 human colon adenocarcinoma cells.	N/A	No modulation to cell viability was detected, however, reduced tumour cell size.	Flow cytometry, tumour size and Q-PCR	Oral and systemic administration of inosine, combined with anti-CTLA-4 and CpG, increased anti-tumour immune responses and reduced tumour size/weight. However, this activity was dependent upon the combined therapy of inosine, anti-CTLA-4, and CpG, and was not replicated in stand-alone treatment of inosine.	^58^
	*In vitro*	Colon cancer	MC38 human colon adenocarcinoma cells	N/A	No modulation to cell viability was detected.	Flow cytometry, tumour size, Q-PCR	The anticancer activity of inosine was dependent upon a combined administration with anti-CTLA-4 or anti-PD-L1 antibodies. This anti-tumour effect was proposed to be mediated primarily *via* T-cell activation.	^58^
**Secondary bile acids**								
Deoxycholic acid	*In vitro*	Liver cancer	HepG2 human hepatic cancer cells	N/A	Inhibited cell viability in HepG2 cell line (LD_50_ = 171 µM/mL).	MTT, Western blot and PCR.	Induced endoplasmic reticulum (ER) stress and TGF-β expression.	^59^
	*In vitro*	Prostate cancer	LNCaP and PC-3 human prostate cancer cells, and RWPE-1 human normal prostate epithelial cells.	N/A	Inhibited cell viability dose-dependently up to a DCA concentration of 200 µM/mL.	Gold electrode-microarray	Exhibited cytotoxic activity in the androgen-dependent LNCaP and the PC-3 cell lines at above 100 µM.	^60^
Chenodeoxycholic acid	*In vitro*	Liver cancer	HepG2 human hepatic cancer cells	N/A	Inhibited cell viability in HepG2 cell line (LD_50_ = 177 µM/mL).	MTT, Western blot and PCR.	Induced ER stress due to changes in intracellular calcium levels and TGF-β expression. Activated caspase-3 and DNA fragmentation, indicating the induction of apoptotic cell death.	^59^
Lithocholic acid	*In vitro*	Breast cancer	MCF-7 and MDA-MB-231 human breast adenocarcinoma cells.	N/A	Inhibited cell proliferation of the MCF-7 (IC_50_ = 104.9 ± 2 μM/mL) and the MDA-MB-231 cells (IC_50_ = 144.8 ± 1 μM/mL).	MTT, flow cytometry, Akt phosphorylation assays, qRT-PCR, Western blot and Lipid (Oil Red O) taining.	Reversion of lipid metabolism deregulation, in addition to inducing apoptosis in cancer cells.	^61^
	*In vitro, In vivo* and *clinical*	Breast cancer	MCF7, 4T1 and SKBR3 breast adenocarcinoma cells. 4T1 xenografted female BALB/c mice.	56 Healthy and 56 breast cancer patients.	Anti-proliferative against (tissue reference concentrations (< 1 μM) breast cancer cells *in vitro* (against the MCF7, 4T1 and SKBR3 cells without affecting primary fibroblast cells) and *in vivo* (4T1 xenograft female BALB/c mice).	SRB assay, propidium iodide assay, Scratch assay, electric cell-substrate impedance sensing, qPCR, HPLC, mass spectroscopy, measurement of oxygen consumption and extracellular acidification rate, SDS-PAGE and Western blotting, immunocytochemistry, infiltration score, tumour-infiltrating lymphocytes calculation, faecal DNA and serum bile acid analyses.	Induced oxidative phosphorylation and the TCA cycle, inhibited epithelial-mesenchymal transition, Vascular Endothelial Growth Factor A expression and boosted antitumor immunity. The molecular mechanism of action of LCA was found to be TGR5 receptor-mediated. Bacterial LCA production was reduced in early-stage breast cancer patients.	^62^
	*In vitro*	Prostate cancer	PC-3 and DU-145 human prostate cancer cells.	N/A	Inhibited cell viability and proliferation of PC-3 (IC_50_ = 32.0 µM/mL) and DU-145 (IC_50_ = 30.4 µM/mL) cells.	WST-1 assay, fluorescence microscopy, SDS–PAGE and immunoblot analysis, gene-silencing using small interfering RNA and ROS assay.	Inhibited cell viability in both cell lines and induced apoptotic and necrotic cell death. Also induced ER stress, mitochondrial dysfunction, and ROS in both cell lines, and autophagy in the PC-3 cell line.	^63^
	*In vitro*	Neuroblastoma cancer	SK-n-MCIXC, BE(2)-m17, SK-n-SH and Lan-1 human neuroblastoma cells.	N/A	Cytotoxic to the neuroblastoma cells with no cytotoxicity or mild cytotoxicity to primary cultures of human neurons in the MTT assay.	MTT, fluorescence microscopy, caspase activity assays, SDS–PAGE, histone H2AX phosphorylation assay	Induced the intrinsic and extrinsic apoptotic pathways in SK-n-MCIXC and BE(2)-m17 cells *via* the initiation of intracellular cascades. Induces non-apoptotic cell death in the Lan-1 cell line, such as necrosis.	^64^
	*In vitro*	Prostate cancer	LNCaP and PC-3 human prostate cancer cells, and RWPE-1 human normal prostate epithelial cells.	N/A	Inhibited cell proliferation in LNCaP (IC_50_ = 40.5 ± 0.07 µM/mL) and PC-3 (IC_50_ = 74.9 ± 0.25 µM/mL) cells.	Gold electrode-microarray, fluorescent microscopy and spectroscopy, and Western blot.	Inhibited cell proliferation in androgen-dependent LNCaP and the PC-3 cell lines with IC_50_ values of 40.5 ± 0.07 µM and 74.9 ± 0.25 µM, respectively. The activated extrinsic pathway of apoptosis (partially dependent on caspase-8 and −3). Enhanced cleavage of Bid and Bax, downregulation of Bcl-2, mitochondrial outer membrane permeabilization and activation of caspase-9. No toxicity against the RWPE-1 human normal prostate epithelial cells.	^60^
	*In vitro*	Colon cancer	HT-29 and HCT-116 human colon adenocarcinoma cells.	N/A	LCA and enantiomer-LCA at 500 μm induced apoptosis in both cell lines compared to the control cells.	DAPI, hexosaminidase assay, Western blot, CD95 immunofluorescence and ROS assay.	Demonstrated morphological signs of apoptotic cell death, including cell shrinkage and cytoplasmic blebbing. Induced apoptosis *via* the activation of caspase-3 and caspase-9. Also inhibited cell proliferation.	^65^
	*In vitro*	Liver cancer	HepG2 human hepatic cancer cells	N/A	Inhibited cell viability in HepG2 cell line (LD_50_ = 66 µM/mL).	MTT, Western blot and PCR.	Induced ER stress and TGF-β expression.	^59^

As bacteriocins have been shown to have a direct impact on gut microbiota in addition to modulating the host immune system, these metabolites may play a key role in the processes of inhibiting carcinogenesis in the intestine as well in determining the efficacy of anticancer treatments and the clinical outcome of cancer. However, there are very limited *in vivo* studies on the anticancer effects of bacteriocins with most studies performed *in vitro*. Additionally, several limitations exist related to the survival and function of bacteriocins *in vivo* as they are mostly dependent on different factors including a) the survival of bacterial strain in the gut, b) specificity of the bacteriocins, and, c) the type of animal model used.^66^ This was further validated in a study that suggested that bacteriocins may not be synthesised or available in high quantities within the GIT, however, there is uncertainty regarding the efficacy of bacteriocin production in existing studies.^68^ Given the inconsistencies with bacteriocin production and availability, the analysis of microbial ecology should be an integral stage of the novel drug discovery process to increase the efficacy of bacteriocin treatment.^68^ The probiotic effects of bacteriocins exemplify the possibility of incorporating these metabolites as novel alternatives to existing antibiotic treatment, as well as pharmabiotics.^69^ The specificity of bacteriocin species ensures that they can target certain pathogens, which is a potential replacement for traditional antibiotics, especially for drug-resistant pathogenic strains.^69^ To investigate this, an *in vitro* study assessed the antibacterial activity of bacteriocins produced by lactic acid bacteria against various strains of the *Helicobacter pylori* species,^70^ which is responsible for a significant number of gastrointestinal cancers. The authors identified that the bacteriocins lacticin A164 and BH5, produced by *Lactococcus lactis*, exhibited the most substantial antimicrobial activity against the *H. pylori* strains, of which the ATCC 43504 strain was the most tolerant of the bacteriocins.^70^ These findings were further supported by another study, which acknowledged that the inhibitory activities of bacteriocins support the use of probiotics in control of *H. pylori* infection and related diseases.^71^ Whilst the structural and functional capacities, as well as immunomodulatory activities of bacteriocin have been well-researched, further studies are required to develop a more comprehensive understanding of the factors that modulate bacteriocin production in the intestinal system. Collectively, based on the available reports, bacteriocins might be promising for the development of novel therapies, especially to target microorganisms that are responsible for carcinogenesis including *H. pylori*.

**Figure 4.**
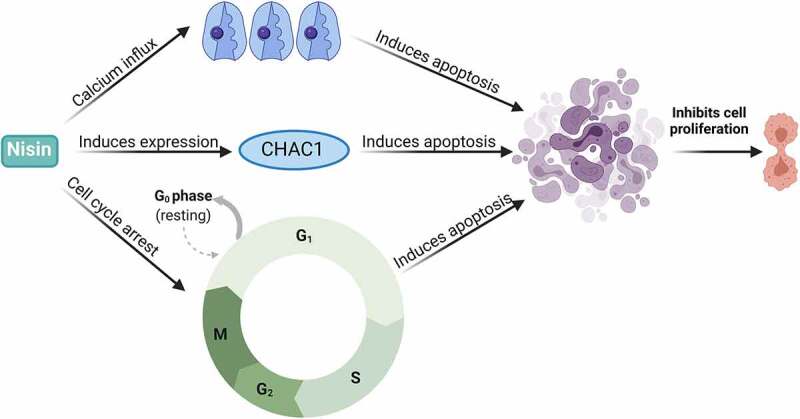
A diagrammatic representation of the molecular mechanisms of action of nisin against cancer cell lines, including the influx of calcium molecules, expression of apoptosis-mediator CHAC1 cation transport regulator, and induction of cell cycle arrest.^15^

Nisin is one of the most explored bacteriocins (also known as lantibiotic) with anticancer potential as evident in several studies on cancer cells. Thus far, four variants of nisin- nisin A, Z, Q and U have been discovered from *L. lactis* and *Streptococcus uberis*.^72^ Nisin is a polycyclic peptide produced by the process of bacterial fermentation known for its antibacterial activity against a broad range of Gram-positive bacteria including *Staphylococcus aureus*, and *Listeria monocytogenes*^73^ and has been studied recently as a potential anticancer peptide against colorectal cancer cells.^13,16,74^ The normal physiological function of nisin is to compete with other Gram-positive bacteria for colonisation of cell surfaces and other cellular resources, which contributes to its prevalent use as a safe food preservative in dairy products.^75^ Due to its safety profile, nisin has been approved in more than 50 countries for use as a food preservative and is generally regarded as safe for humans by the World Health Organisation.^67^ Recent studies have explored its use in the inhibition of tumorigenesis in head and neck squamous carcinoma cells both *in vivo* and *in vitro*.^16^ Nisin has been shown to increase the apoptotic index in cancer cell lines *via* the intrinsic apoptotic pathway.^13^ This was further supported by another study that observed that increasing concentrations of a specific nisin variant correlated with increasing levels of apoptotic cancer cell death and a decrease in cell proliferation of head and neck cancer cells.^16^ It was earlier proposed that nisin exerted these cytotoxic effects on cancer cells through CHAC1 (ChaC Glutathione Specific Gamma-Glutamylcyclotransferase 1), which is a pro-apoptotic cation transport regulator and is considered an apoptotic mediator in relation to tumorigenesis.^15^ Nisin-induced CHAC1 expression increased calcium influxes and induced cell cycle arrest in the G_2_ phase which led to apoptosis and a decrease in tumour cell proliferation (Figure 4).^15^ However, the authors also acknowledged that the optimal therapeutic dose must be determined for the potential use of nisin in cancer therapy which is plausible based on the history of safe human consumption of nisin.^15^ Furthermore, an *in vivo* study on the association of nisin ZP against head and neck squamous cancer cells (HNSCC) demonstrated that nisin ZP reduced tumorigenesis in mice models, and long-term treatment with nisin ZP extended the survival of the mice with normal organ histology ^16^ (Table 1). This was further supported by another *in vivo* study, in which nisin decreased tumorigenesis of HNSCC through the induction of apoptosis *via* upregulating the CHAC1 gene expression.^15^ The same study showed that nisin at 80 μg/mL inhibited the proliferation of the UM-SCC-17B HNSCC cells *via* cell cycle arrest in the G_2_ phase after 24 h.^15^ Recent studies have also cloned and expressed the fusion protein derived from the three bacteriocin- nisin, enterocin, and epidermicin in *E. coli* to explore the possibility of utilising the fusion protein for gastric cancer.^76^ Additionally, nisin has been shown to inhibit the proliferation of blood, breast, brain, colon, gastrointestinal, liver and skin cancer cells pre-clinically mostly through induction of apoptosis as shown in Table 1. Despite its promising anticancer activity and safety profile as depicted in these studies, nisin has not been evaluated alone or in combination with standard anticancer therapy clinically. Due to its selective toxicity towards cancer cells as compared to normal cells,^67^ further clinical studies should be performed to explore the therapeutic potential of nisin. Current literature has demonstrated the potential use of bacteriocins in conjunction with standard chemotherapeutic drugs as an alternative approach to cancer treatment. A recent study identified that the application of nisin and 5-FU as a combination therapy exhibited synergistic activity against 7,12-dimethylbenz(a)anthracene-induced skin cancer *in vivo* and lowered their IC_50_ values by an eight-fold against the A431 epidermoid carcinoma cells with a combination index value of 0.188.^77^ The anticancer activity of the combination was mediated by modulation of apoptotic, angiogenic and cell proliferative pathways with significant reduction of tumour size and number (mean tumour volume and mean tumour burden) compared to the mono treatments in that study.^77^ Furthermore, within the food and pharmaceutical industries, bacteriocins have been observed to be capable of replacing antibiotics which could assist in combatting multi-drug resistant pathogens.^24^ This approach might also be useful in eliminating carcinogenic pathogens from the gut. However, further research is necessary to understand the efficacy of bacteriocins in anticancer therapy both as mono and combination regimens with standard chemotherapy.

## Short-chain fatty acids (SCFAs)

**Figure 5.**
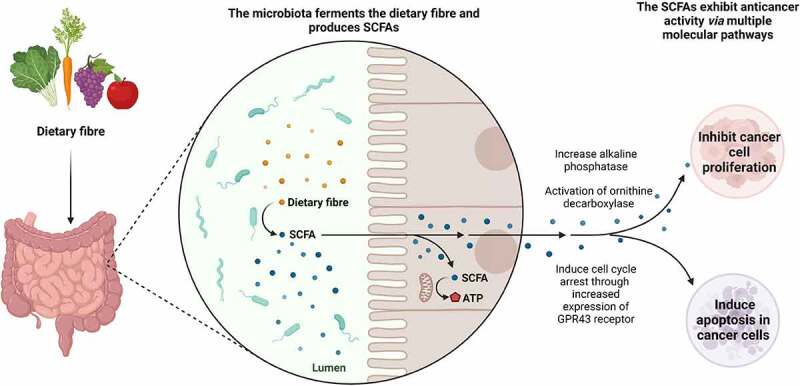
A diagrammatic representation of the synthesis of short-chain fatty acids (SCFAs) *via* the fermentation of dietary fibre by gut microbial species, and the anticancer action of these metabolites through different molecular mechanisms.

SCFAs have been well-researched in recent years for their inhibitory effects on, especially colon and breast cancer cells (Table 1). It has been established that dietary nutritional factors play a substantial role in the progression of CRC, and the absence of certain factors can disturb metabolic and homeostatic pathways within the intestinal system that in turn promote tumourigenesis.^78–80^ SCFAs are derived from the breakdown of dietary fibre, in which fermentation of the non-digestible carbohydrates occurs in the lower gastrointestinal system, the cecum and large intestine, by anaerobic cecal and colonic microorganisms.^81^ This fermentation process results in a group of metabolites with SCFAs as the primary metabolites.^81^ The predominant bacterial species responsible for producing SCFAs are the *Faecalibacterium prausnitzii, Clostridium leptum, Eubacterium rectale*, and *Roseburia* species, as well as lactate-utilising species that synthesise SCFAs from lactate and acetate, including *Anaerostipes* species and *Eubacterium hallii*.^82^ A profiling study on CRC patients observed that the dominant group of *Bifidobacterium* species disappeared, and different spectrums of *Bifidobacterium* was present in the CRC patients (n = 14) compared to the non-CRC participants (n = 14). This observation correlated with significantly lower SCFA levels in the CRC patients compared to the non-CRC group.^83^ The same research group earlier demonstrated that CRC patients (n = 14, Indonesian citizens, 18 years of age or older) were presented with lower levels of acetate, propionate, and butyrate, than the non-CRC participants (n = 14, Indonesian citizens, 18 years of age or older), indicating the indirect contribution of SCFAs in the prevention of CRC development.^84^ Several key SCFAs have exhibited protective action against colon carcinogenesis, including butyrate, acetate, and propionate, which are synthesised *via* anaerobic bacterial and carbohydrate fermentation^21,79,85,86^ (Figure 5). In particular, the major health benefits of fibre consumption are attributed to the production of SCFAs through the fermentation processes occurring in the colon.^79^ At the molecular level, butyrate has been observed to inhibit cell proliferation and induce apoptosis and cell differentiation through the initiation of histone hyperacetylation in cancer cells.^5,78,86,87^ Furthermore, SCFAs can recognise G protein-coupled receptors- GPR41, GPR43 and GPR109A on the surface of colonocytes and immune cells including macrophages and T cells which in addition to histone hyperacetylation leads to an enhancement in total colonic regulatory T cell numbers and the levels of the anti-inflammatory cytokines interleukin-10 (IL-10) and transforming growth factor-β (TGFβ) (Figure 1).^5^

**Figure 6.**
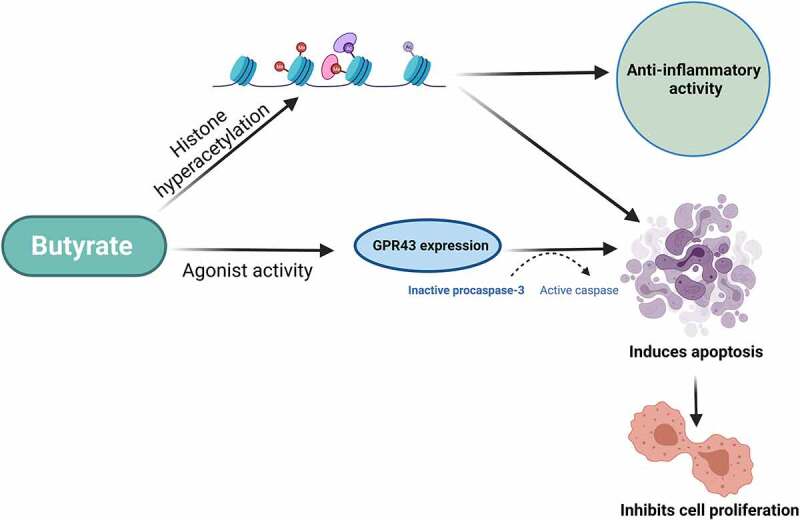
A diagrammatic depiction of the biological activities of butyrate on cancer cells *via* G protein-coupled receptor 43 (GPR43) and modulation of immune cells.^32,86,87^

A study investigated the effects of three SCFAs- butyrate, acetate, and propionate on the growth of the HT29 human colorectal adenocarcinoma cells and showed that butyrate and propionate were more effective in inhibiting the growth of HT29 cells, in comparison to acetate which had no observable effect.^85^ Similar observations were made in another study^86^ where butyrate, propionate and valerate (a type of SCFA) inhibited the human colon carcinoma cells with no activity reported for acetate and caproate (a type of SCFA). Butyrate in this study also significantly increased apoptosis in the cancer cells.^86^ The inhibitory effect of butyrate and propionate on the proliferation of cancer cells was associated with the activation of ornithine decarboxylase, which is an important enzyme of polyamine metabolism, however, it was also noted that these SCFAs did not act solely on the polyamine pathway.^85^ Data from that study revealed that butyrate and propionate caused an increase in alkaline phosphatase activity indicating that they may play an important role in the normal physiology of the colon and could also be a contributing factor in the protective influence of dietary fibres on colon carcinogenesis.^85^ The tumour suppressive functions of SCFAs, most particularly butyrate, are believed to be caused by the histone hyperacetylation-mediated pathway which results in the conversion of inactive procaspase-3 to catalytically active protease (apoptotic) (Figure 6).^32,86,87^ This anti-tumour effect of SCFAs has also been supported by epidemiological studies that highlight a fibre-rich dietary lifestyle correlates with the reduction of CRC risks.^32,88^ Other health benefits of butyrate dietary supplementation include the prevention of insulin resistance and obesity induced by a high-fat diet, which is achieved through a decrease in adiposity and an increase in insulin sensitivity in peripheral tissues.^79^ This was further supported in a mice model study that observed that the administration of butyrate as a dietary supplement prevented and treated diet-induced insulin resistance.^89^ The authors also acknowledged that the mechanism of action of butyrate was directly associated with the induction of mitochondrial activity and the promotion of energy expenditure, which was a significant development in understanding the anticancer mechanisms of butyrate.^89^

As aforementioned, the introduction of butyrate into cells leads to histone hyperacetylation *via* the inhibition of histone deacetylase activity.^5,78,86,87^ Therefore, butyrate may serve as a key factor in determining the role of histone acetylation in the structure and function of chromatin, demonstrating a strong association between butyrate and histone deacetylase inhibitors in preventing and managing cancer cell proliferation.^90^ The mechanism of action of this inhibitory activity has also been studied and showed that there is a close association between the SCFA-mediated activation of the GPR41/GPR43 receptor signalling pathways and the inhibition of histone deacetylases.^91^ GPR43 is recognised by SCFAs, and expression of this receptor is found predominantly in the large intestine and haematopoietic tissues and is frequently lost in colon cancer cell lines.^32^ It has been identified that restoration of GPR43 receptor expression in the HCT8 human colonic adenocarcinoma cells led to an increased apoptotic cancer cell death following G_0_/G_1_ cell cycle arrest.^32^ In particular, the treatment of the HCT8 cancer cells with butyrate and propionate led to an increase in the GPR43 receptor expression and apoptotic cell death (Figure 6).^32^ Therefore, it has been speculated that the GPR43 receptor serves as a functional tumour suppressor to mediate the apoptotic effects of SCFAs in CRC.^32,85,92,93^ This was further supported by another study that observed agonist activity for both butyrate and propionate on the GPR43 receptor, in which the highest concentrations of GPR43 were identified in immune cells under certain pathophysiological conditions.^92^ Another report also suggested that SCFA-mediated growth arrest in colon carcinoma cells requires the *p21* gene as SCFAs were ineffective against the *p21*-deleted HCT-116 colon cancer cells. In normal cells, p21 functions as a cell cycle inhibitor and anti-proliferative effector, whereas in some cancers it is dysregulated.^94^ The role of p21 in cancer has been established in several reports as a tumour-suppressor protein under the p53 transcription factor activity.^94^ The complex interplay among gut microbiota, the immune system and dietary factors have been researched in recent years.^4^ Whilst more investigations are required to adequately determine the association between SCFAs and the immunometabolism of T cells, including specific metabolic targets, it has been shown that diets rich in SCFAs have displayed suppressive action on T cell-mediated autoimmune responses, which may be achieved *via* the regulation of cytokine expression and T cell function by these secondary metabolites.^91^ Despite the promising anti-tumoural action of butyrate identified in existing studies, research has also observed its pro-tumoural effects in the development of CRC. A study assessing the impacts of pathogenic bacteria on CRC development acknowledged that the pro- or anti-tumoural effects of butyrate are dependent on multiple factors, including duration and amount of exposure to the treatment, and the studied cell type.^95^ That study coined the term ‘butyrate paradox’, where the effects of butyrate are determined by its concentration, with low levels promoting tumorigenesis and high levels inhibiting tumour.^95^ Another study validated the ‘butyrate paradox’ by utilising a mice model and observed that, despite the evident anti-tumoural activity of butyrate, low concentrations of butyrate promoted the development of CRC by increasing the proliferation of colonic epithelial cells.^5^ Many studies have observed similar carcinogenic effects of butyrate in animals and humans, however, further investigation is required to understand the interactions between the host’s genetics, microbial composition, and presence of other gut metabolites to constitute this paradoxical effect.^96^ The low levels of butyrate have also been shown to initiate a pro-inflammatory environment within the host that disrupt the gut microbial composition by suppressing potential pathogens and encouraging colonisation by butyrate-producing species.^5^ The complexity of butyrate activity dependent upon concentration is a vital consideration in its use as a potential anticancer therapeutic. In relation to standard chemotherapeutic drugs, one study identified that butyrate significantly improved the efficacy of 5-fluorouracil (5-FU) against colon cancer cells and increased impairment of DNA synthesis caused by 5-FU.^97^ Parallel observations were made in a previous study that reported that forming a conjugate targeted delivery system with a standard chemotherapeutic drug such as doxorubicin, and a SCFA could improve the efficacy of the standard drug, limit the occurrence of drug resistance, and more efficiently target the tumour microenvironment.^98^ Through the inhibition of histone deacetylase (HDAC), butyrate might be beneficial in improving the clinical efficacy and reducing the toxicity of standard chemotherapy.^99^

Overall, SCFAs are promising specifically in the context of colon cancer. Future studies should evaluate the effects of SCFAs on other cancer types including pancreatic and gastric cancer to understand their molecular mechanisms of action. Studies should also explore the impact of SCFAs on the efficacy and safety of standard chemotherapy and the prognosis of cancer.

## Phenylpropanoid-derived metabolites

Phenylpropanoid-derived metabolites, such as phenolic acids, are a significant component of plant secondary metabolism and have been demonstrated to inhibit the growth of different cancer cell types in several studies through a number of molecular pathways.^100–103^ As a derivative of plant secondary metabolism and a constituent of diets rich in plant foods, phenylpropanoids have exhibited chemopreventive, antioxidant, anti-inflammatory, and antimitotic activities in the host.^104–106^ The biosynthesis of phenylpropanoids has been extensively researched in the past decade to develop a greater scientific understanding of the upstream and downstream enzymes responsible for the development of these secondary metabolites.^104^ It has been speculated that specialised phenylpropanoid products can be developed from the recognised mechanistic foundations of phenylpropanoid metabolising enzymes, which can include diverse novel compounds with both dietary and medicinal properties in human health.^104^ One study on human faecal samples found that phenylpropanoid-derived compounds, including phenylacetic acid and 4-hydroxylphenylacetic (the two most abundant metabolites detected) were synthesised from both plant-rich diet and the microbial fermentation of AAAs in the colon.^21^ In particular, phenylpropanoids-derived compounds such as phenylacetic acid (PAA) and 4‐hydroxylphenylacetic acid (4‐hydroxyPAA) are produced through microbial fermentation of AAAs- phenylalanine, tyrosine and tryptophan in the colon by *Bacteroidetes (Bacteroides thetaiotaomicron, Bacteroides eggerthii, Bacteroides ovatus, Bacteroides fragilis, Parabacteroides distasonis)*, and Firmicutes (*Eubacterium hallii* and *Clostridium bartlettii*).^21^ A profiling study of colorectal cancer patients detected increased levels of *Bacteroides fragilis* in patients with advanced diagnoses (stage III and IV CRC), and these levels were greater in the colon than in the rectum.^107^ This study involved patient participation from two cohorts (average age 59 years with an equal division of gender); the first cohort consisted of 55 paired CRC patient samples with no pre-selected condition in addition to CRC, and the second cohort consisted of 18 patients that also had been diagnosed with sporadic microsatellite instability.^107^ This increased presence of bacteria responsible for producing phenylpropanoid-derived metabolites was indicative of the potential causative association between advanced progression of the tumour and increase in anti-tumoural bacteria and metabolite production.^107^ An *in vitro* study on probiotic metabolites of *Lactobacillus rhamnosus* observed that 4-hydroxyPAA activated mitochondrial-regulated apoptosis and induced cell proliferation against the HepG2 liver cancer cell line.^108^ Similarly, an *in vitro* study examined the activity of a novel Zn(II) complex combining phenylacetic acid and the 4,4’-bipyridine ligand, in which the complex was identified to inhibit the HeLa cervical cancer cell line through induction of apoptosis.^109^

**Figure 7.**
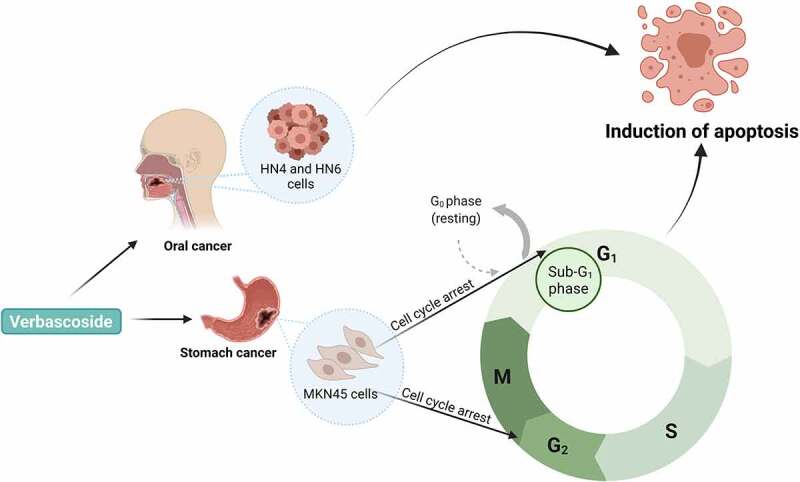
A schematic representation of the molecular mechanisms of action of verbascoside, a gut microbial metabolite, on the HN4 and HN6 human oral squamous cell carcinoma and the MKN45 gastric epithelial cancer cells.^45,47^

Phenylpropanoids are well-known for their microbial diversity and biosynthetic origins, however, further studies are required to better understand the mechanisms of action and antimicrobial activities of these compounds,^110^ especially against carcinogenic bacteria and viruses. Dietary verbascoside, a phenylpropanoid compound, has been shown to influence gut morphology due to its antimicrobial and antioxidant properties.^111^ The authors demonstrated that verbascoside protected the gastrointestinal tract from oxidative stress with potential appetite-stimulatory effect *via* modulation of the protein expression of the gastrointestinal taste receptors.^111^ Verbascoside has also been found to arrest the MKN45 gastric epithelial cancer cells at the sub-G_1_ and G_2_/M phases of the cell cycle (Figure 7).^47^

A study showed that human and rat gut microbiota can break down acteoside, a type of verbascoside into 14 metabolites including 8 degradation metabolites, 2 isomers in intestinal bacteria and intestinal enzyme samples and 4 parent metabolites.^112^ Acteoside also exhibited a significant anti-inflammatory effect by inhibiting LPS-induced PGE_2_, nitric oxide and TNF-α in mouse peritoneal macrophages in a concentration-dependent manner.^113^ Whilst further research is required to understand the anti-inflammatory effects of these compounds, it has been suggested that polyphenols target multiple inflammatory components and modulate immune processes *via* the synthesis of proinflammatory cytokines, immune cell regulation, and gene expression.^114^ These biologically active compounds promote extended health benefits for several chronic inflammatory diseases, including cancers, by supporting the immune system and preventing the onset of chronic disease as observed in preclinical experimental models and clinical studies.^114^ In recent years, research has prioritised focusing on the use of natural substances that are most cost-effective and present with fewer adverse effects.^115^ Existing studies have also assessed the combined implementation of phenylpropanoids and standard chemotherapeutic agents in the treatment of cancer. An *in vitro* study aimed to assess the combined therapeutic potential of eight distinct phenylpropanoids in conjunction with 5-FU against the HeLa cervical cancer cells and identified that eugenol, ferulic acids, and caffeic acids demonstrated synergy when combined with 5-FU.^116^ Additionally, phenylpropanoids exhibited minimal haemolytic activity on human erythrocytes supporting the use of these compounds as pharmaceutical drugs without causing toxicity within the host.^116^ Despite their broad spectrum of biological properties against cancer as well as in the modulation of gut microbiota, further studies are required to understand the metabolic and catabolic pathways of phenylpropanoid by the gut microbiota and the mechanisms by which these compounds modulate inflammatory and microbial processes.^117^

## Prenylflavonoids

Xanthohumol (XN) is a prenylated flavonoid found in hops and it has shown promising anticancer activity in recent years.^52^ The gut microbiota can metabolites XN to produce 8-prenylnaringenin (8-PN), a very potent phytoestrogen.^52^ Primarily, 8-PN has demonstrated strong anticancer action across multiple cancer cell lines (Table 1) and has been acknowledged for other health benefits.^49,118^ The bioactivity of 8-PN might be attributed to its greater oral bioavailability in healthy individuals than its isomer 6-PN, despite a clinical trial demonstrating that both compounds had a similar effect on increasing cell viability of peripheral blood mononuclear cells.^118^ A study assessed the anticancer activity of 8-PN *in silico* and *in vitro* against the SK-MEL-28 and BLM human metastatic melanoma cells and observed that it mediated anticancer action *via* the inhibition of HDAC.^49^ An earlier study also showed that 8-PN inhibited the proliferation of the MCF7 human breast cancer cells through induction of apoptosis.^51^ The proposed mechanism of action of this activity was the increased proliferation of estrogen-responsive cells by the 8-PN metabolite, through the interference with the estrogen receptor-associated PI3K pathway.^51^ The strong estrogenic action of 8-PN was also observed in other studies where it showed greater activity than that of other established phytoestrogens, including genistein, daidzein, and coumestrol.^119^ Another study compared the anticancer and apoptotic potential of 8-PN with other side-chain variants of prenylflavanones and found that 8-PN could target multi-drug resistant leukaemia cells and induced mitochondria-dependent apoptosis.^120^ The *in vitro* action of 8-PN on various stages of colorectal tumourigenesis was also investigated.^48^ This metabolite inhibited cell proliferation of the HT115 cells in a dose-dependent manner, with a growth reduction of up to 46%, in comparison to the untreated control.^48^ The authors demonstrated that 8-PN exerted the anticancer activity at various key stages of colorectal tumourigenesis, which could be beneficial to improve the poor prognoses of CRC.^48^ A more recent study specifically assessed the anticancer activities of 8-PN against HCT-116 colon cancer cells and determined that it conferred anti-proliferative activity *via* the induction of extrinsic and intrinsic pathway-mediated apoptosis.^50^ These *in vitro* findings of the anticancer activity of 8-PN warrant further *in vivo* and clinical studies to examine its mechanisms of action and potential use as a natural anticancer agent.

## Natural purine nucleosides

Natural purine nucleosides have demonstrated anticancer potential *via* different molecular mechanisms. The efficacy of nucleoside-based anticancer drugs is determined by the cellular transporters that modulate the movement of drugs into and out of the cell.^121^ Analyses of different parasitic species have identified two prominent purine nucleoside transporters- an adenosine transporter and an inosine transporter.^122^ Inosine is a metabolite synthesised by the catabolism of the adenosine compound, which exhibited diverse anti-inflammatory and immunomodulatory effects *in vivo* by acting directly on adenosine receptors.^123^ The proposed modulatory mechanism of action of these effects is through the adenosine A_2A_ receptor (A_2A_R), in which inosine-regulated activation of A_2A_R initiates cAMP production and extracellular signal-modulated phosphorylation of kinase-1 and −2.^123^ The findings of an *in vivo* study identified that inosine initiates ERK1/2-biased signalling as an agonist, in which it can amplify and extend A_2A_R activation, and this has significant pharmacological implications.^123^ Another *in vivo* study on the inosine metabolite acknowledged that it enhanced T cell antitumour activity in colorectal, bladder, and melanoma cancer types while amplifying the effects of checkpoint blockade immunomodulation.^58^ The authors also confirmed that the bacteria *Akkermansia muciniphila*, associated with responsiveness to immune checkpoint blockade (ICB) therapy in humans, used inosine- A_2A_R signalling for its ICB-promoting effect.^58^ The proposed mechanism of action of inosine on bladder cancer was the enhancement of the function of anti-CTLA-4 to increase infiltration of IFN-ɣ^+^CD4^+^ and IFN-ɣ^+^CD8^+^ T-cells into the tumour, as well as reducing overall tumour weight when administered with CpG (DNA oligonucleotides containing unmethylated deoxycytidylyl-deoxyguanosine dinucleotides^124^) as a combination therapy.^58^ The study also reported that inosine produced by *Bifidobacterium pseudolongum* increased the activation of a cDC-dependent T_H_1 cell circuit, which enhanced the overall effect of ICB therapies in mouse models of intestinal and epithelial tumours.^58^ Whilst inosine has presented with promising anticancer action in combination with other immunotherapies, further investigation is required into its stand-alone anticancer activity and mechanisms of action within the host.

## Secondary bile acids

### Carcinogenic activity

Secondary BAs are metabolised from the dehydroxylation of primary bile acids by anaerobic bacteria in the large intestine, in which primary BAs are originally synthesised from cholesterol in the liver hepatocytes prior to being released into the intestinal system.^125,126^ The bacterial species primarily involved in the production of secondary bile acids are members of the *Clostridium* genus including *C. scindens, C. hiranonis, C. hylemonae*, and *C. sordellii*.^127^ Unlike SCFAs, BAs have been shown to exhibit pro-carcinogenic activity. Studies have demonstrated that the exposure of the gastrointestinal (GIT) tract cells to high levels of secondary BAs are a major contributing risk factor towards the development of GIT cancers and a high level of BAs is most commonly seen in individuals with a high dietary fat intake.^12,125,128,129^ High-fat diets lead to an increase in the levels of secondary BAs in the enterohepatic circulation, including deoxycholic acid (DCA) and lithocholic acid (LCA), both of which can be risk factors for the induction of inflammation and cancer in the colon.^11^ This accumulation of secondary BAs, particularly DCA, is due to the incapability of the human liver in returning 7α-hydroxylating secondary BAs *via* the portal vein, causing the high-level accumulation in humans consuming a ‘Western diet’.^127^ It has been well-established that DCA is capable of initiating cell-signalling pathways involved in the onset of various diseases.^127^ To support this, a clinical study profiling metabolite concentrations in CRC patients observed an increased DCA level in the faeces, blood serum, and bile of participants.^130^ Similarly, it has also been reported that mice fed with a high-fat diet presented with higher levels of the *C. sordellii* compared to other microbial species suggesting its role in increasing the DCA levels.^127,131^ Excessive exposure to BAs can further lead to the generation of ROS with subsequent disruption of the mitochondrial and cell membrane as well as DNA damage.^12^ The oxidative and DNA damage-related stress caused by prolonged exposure of cells to BAs initiates genomic instability within the cells, leading to the development of apoptotic resistance and the eventual onset of cancer.^12,129^ It has been reported that nuclear receptors are directly associated with the modulation of BA metabolism and detoxification as they operate as transcription factors in the protection from the tumour promoting action of secondary BAs.^132^ This is a significant factor of consideration in circumstances of human genetic mutational predispositions, in which secondary BAs can accelerate the intestinal adenoma-adenocarcinoma sequence through the initiation of the Wnt/β-catenin signalling pathway.^133^ The genetic and environmental factors associated with the pro-carcinogenic activity of secondary BAs provide a foundation for further investigation into the role these metabolites play in the origin and prognosis of cancer.

As a common secondary BA present in individuals with a fat-rich diet, DCA functions as a significant environmental trigger in the onset of CRC.^134^ Whilst the exact mechanism of action of DCA on intestinal tumorigenesis requires further investigation, it has been observed that it disrupts the intestinal mucosal barrier and increases pro-inflammatory cytokine production in the intestine, which is a key precursor to the development of intestinal cancer.^134^ Gut microbial communities are inherently responsible for the modulation of intestinal homeostasis, in which dysbiosis to the microbiome is directly associated with intestinal tumorigenesis that is induced by high concentrations of DCA in the intestine.^133^ In an *in vivo* study, the DCA-treated mice presented with alterations to the intestinal microbiome composition, which was coupled with an impaired intestinal barrier, inflammatory processes, and tumorigenesis.^133^ The findings of that study provided evidence that the introduction of DCA into the intestine led to disturbances in the gut microbial composition and promoted carcinogenesis in the intestine. These findings, in correspondence with other available studies, emphasised that the interactions between secondary BAs and gut microbiota were responsible for the initiation of intestinal carcinogenesis, which is important for developing novel therapeutic strategies especially for GIT cancers.^133,135^ The hyperproliferation of the colon mucosa is a preliminary stage in the progression of colorectal carcinogenesis and in animal models, DCA was observed to stimulate the proliferation of colorectal epithelial cells with a tumour promoting activity.^136^ Another study assessed the effects of DCA on the migration of the Caco-2 human colon cancer cells and found that the migration of the cancer cells was likely associated with protein kinase C.^137^ The tumour promoter activity of DCA was further validated in another study showing that DCA could activate protein kinase C and phospholipase C *via* increased Ca^2+^ entry at the plasma membrane of the BHK-21 fibroblast cells.^138^ A biopsy study on 19 patients with and without colon cancer in 1999 demonstrated that a significant increase in colorectal proliferation was correlated with the serum DCA levels but not with the serum levels of other BAs such as lithocholic, cholic, chenodeoxycholic, and ursodeoxycholic acid.^136^ More human interventional studies are prudent to examine whether a decrease in DCA levels lowers the risk of carcinogenesis. It has been demonstrated that a higher risk of CRC in the American population was likely in part due to their high-fat and high-protein diet, which leads to the promotion of microbial species that can produce potentially carcinogenic secondary BAs.^80^ Similarly, a more recent investigation identified that there are multiple mechanisms of action and a diverse range of signals involved in the promotion of CRC development by BAs and their derivatives, which highlights the potential of targeting primary and secondary BAs in the prevention of CRC.^139^ However, to date, there are limited studies exploring these mechanisms and signalling pathways, which emphasises the need for future preclinical and clinical research on BAs to assist in the prevention and treatment of cancers.

### Anticancer activity

Despite the predominantly carcinogenic nature of secondary BAs, lithocholic acid (LCA), a derivative of cholic acid, demonstrated anti-proliferative action on different cancer cell lines (Table 1).^61–64,140^ *In vitro* studies on the human breast adenocarcinoma (MCF-7 and MDA-MB-231), human prostate cancer (PC-3, LNCaP and DU-145), hepatic cancer (HepG2) and neuroblastoma cancer (BE(2)-m17, SK-n-SH, SK-n-MCIXC and Lan-1) cells have found that LCA inhibited the cancer cell growth by different molecular mechanisms of action (Table 1).^59–61,63,64^ Notably, LCA inhibited the growth of the human prostate cancer (LNCaP and PC-3) cells through caspase-3, 8 and 9 mediated apoptosis.^60^ LCA was also able to induce endoplasmic reticulum (ER) stress and transforming growth factor-β (a potent profibrogenic factor that induces apoptosis of hepatocytes and liver fibrosis) in the HepG2 liver cancer cells.^59^ The cytotoxicity and ER stress in the HepG2 liver cancer cells were largely dependent on the hydrophobicity of the secondary BAs, with chenodeoxycholic (a hydrophobic secondary BA) exhibiting the greatest activity among the tested secondary BAs.^59^ Another study^62^ demonstrated that LCA is anti-proliferative against breast cancer cells *in* *vitro* (against the MCF7, 4T1 and SKBR3 cells without affecting primary fibroblast cells) and *in vivo* (4T1 xenograft female BALB/c mice) and induced oxidative phosphorylation and the TCA cycle, inhibited epithelial-mesenchymal transition, vascular endothelial growth factor A expression and boosted antitumor immunity. The molecular mechanism of action of LCA was found to be TGR5 receptor-mediated.^62^ The authors also reported that in early-stage breast cancer patients, bacterial LCA production was reduced.^62^ Despite these new findings on the anticancer activity of some secondary BAs, its in-depth mechanisms of mitochondrial dysfunction and cytotoxicity in cancer cells are yet to be confirmed and requires further investigation.

## Conclusion and future directions

The vital role of the gut microbiota in the maintenance of gut homeostasis and immune health has been well-documented in the literature. This acknowledgement led to the investigation into the influence of gut microbial metabolites on human health, most specifically in relation to cancer. The complex cross-talk between specific gut microbial metabolites and the progression or inhibition of cancer cell growth is an emerging area of anticancer research. Studies conducted *in vitro* and *in vivo* have been crucial in understanding the anti- and pro-cancer activity of these gut metabolites in the host. For instance, gut metabolites including SCFAs and bacteriocins have been increasingly reported to have cytotoxic activity on various cancer cell types. SCFAs are commonly known for their antioxidant and anti-inflammatory action on host health, which help in inhibiting cancer cell proliferation. Furthermore, SCFAs have been shown to be a key player in the inhibition of obesity-induced insulin resistance, which is an important consideration in the development of colorectal cancer. Similarly, bacteriocins displayed anti-tumour potential *via* direct (by induction of the apoptotic pathway in cancer cells) and indirect (by conferring colonisation resistance on epithelial surfaces to inhibit pathogenic microbes) actions. In addition, phenylpropanoid-derived metabolites are observed in high concentrations in individuals that consume a plant-rich diet and have been extensively researched over the past decade for their antioxidant, anti-inflammatory, and inhibitory activities on cancer cell proliferation. Comparatively, secondary BAs have been found to express pro-carcinogenic activity in the host. These toxic by-products can initiate genetic instability within the cell and encourage apoptotic resistance increasing cancer cell growth.

Despite the current evidence on the anticancer potential of gut metabolites such as SCFA, bacteriocins and phenylpropanoid-derived compounds, most of these studies were conducted *in vitro* with limited *in vivo* testing and no reported clinical studies. The mechanisms of action of these gut microbial metabolites are yet to be comprehensively understood in the context of carcinogenesis and anticancer activity. Further investigation will be crucial to determine the appropriate therapeutic dose of the gut metabolites for their safe clinical use in anticancer therapy. Therefore, future research should prioritise studies- a) to further understand the molecular mechanisms of action and b) to evaluate the potential toxicity of these metabolites, and c) to determine the therapeutic dose needed. Furthermore, it would be exciting to evaluate if these gut metabolites interact favourably with standard chemotherapies to increase their efficacy and safety in clinical settings.
